# Airborne Transmission of Avian Origin H9N2 Influenza A Viruses in Mammals

**DOI:** 10.3390/v13101919

**Published:** 2021-09-24

**Authors:** C. Joaquín Cáceres, Daniela S. Rajao, Daniel R. Perez

**Affiliations:** Department of Population Health, College of Veterinary Medicine, University of Georgia, Athens, GA 30602, USA; cjoaquincaceres@uga.edu

**Keywords:** H9N2, influenza, aerosol, interspecies, mammals, zoonotic, pandemic

## Abstract

Influenza A viruses (IAV) are widespread viruses affecting avian and mammalian species worldwide. IAVs from avian species can be transmitted to mammals including humans and, thus, they are of inherent pandemic concern. Most of the efforts to understand the pathogenicity and transmission of avian origin IAVs have been focused on H5 and H7 subtypes due to their highly pathogenic phenotype in poultry. However, IAV of the H9 subtype, which circulate endemically in poultry flocks in some regions of the world, have also been associated with cases of zoonotic infections. In this review, we discuss the mammalian transmission of H9N2 and the molecular factors that are thought relevant for this spillover, focusing on the HA segment. Additionally, we discuss factors that have been associated with the ability of these viruses to transmit through the respiratory route in mammalian species. The summarized information shows that minimal amino acid changes in the HA and/or the combination of H9N2 surface genes with internal genes of human influenza viruses are enough for the generation of H9N2 viruses with the ability to transmit via aerosol.

## 1. Introduction

Influenza A viruses (IAV) are members of the family Orthomyxoviridae with a segmented RNA genome of negative polarity. IAV are divided into subtypes by the combination of the surface proteins, the hemagglutinin (HA, H1–H18) and the neuraminidase (NA, N1–N11) [1]. The natural hosts of IAV are wild aquatic birds, particularly waterfowl and seabirds, in which most of the IAV subtypes have been described [2,3]. IAVs sporadically spill over between the wild bird reservoir and domestic poultry species and may lead to disease outbreaks. This has been the case for some H5Nx, H7Nx or H9N2 IAV viruses [4,5,6,7,8,9]. IAVs of avian origin are classified into high pathogenicity avian influenza viruses (HPAIVs) and low pathogenicity avian influenza viruses (LPAIVs) based on the pathotype in chickens and/or the presence of a polybasic amino acid cleavage site composed of Arginine (R) or Lysine (K) [10]. The HA’s polybasic R/K cleavage site of HPAIVs allows for processing of the HA by endogenous cellular furin-like proteases, leading to systemic infections and increased pathogenicity. In contrast, LPAIVs contain no more than a tri-basic cleavage site in the HA, making them dependent on extracellular trypsinlike proteases for processing, mostly limiting infections to sites where such enzymes are abundant (e.g., respiratory and/or gastrointestinal systems) [10,11,12]. However, LPAIVs have been shown to replicate in the oviducts and kidneys of some infected birds, suggesting the presence of proteases in other tissues that may be able to process monobasic cleavage sites [13,14,15]. Recent H9N2 isolates from Asia and Middle East have increasingly shown the presence of a tri-basic HA cleavage site, which was associated with increased pathogenicity and transmission in chickens in comparison with viruses with mono-basic cleavage sites [16]. However, these viruses with the tri-basic cleavage site did not demonstrate a HPAI phenotype but rather an intermediate step towards a gain in pathogenicity, and were still considered LPAIV. Only viruses of the H5 or H7 subtypes have been associated with the HPAI pathotype.

### 1.1. H9N2 Avian Influenza Viruses

IAVs of the H9N2 subtype are widespread in several species of wild waterfowl, shorebirds, and poultry, such as chickens, turkeys, and quails, among others [17,18,19,20,21,22]. H9N2 IAVs are the most prevalent LPAIVs, and are enzootic in poultry in parts of Asia, the Middle East, and Africa (reviewed in [3]). H9N2 IAVs were first isolated from turkeys in Wisconsin, USA in 1966 [23], with subsequent sporadic detections in poultry in the US. H9N2 viruses were first isolated from healthy ducks from farms and live poultry markets in Hong Kong between 1975 and 1985 [24,25]. In 1988, the first evidence of H9N2 infection in poultry in Asia was reported after a respiratory outbreak in quails [26]. Currently, H9N2 IAVs are widespread in poultry species around the world with particularly high prevalence in Asia [7,27,28]. Recently, H9N2 IAVs have also been detected in Sub-Saharan Africa, historically considered a cold spot for animal IAV [29].

H9N2 IAV infections can be mild. However, significant economic losses are associated with H9N2 IAV infections because of delayed growth and lower egg production. Studies have shown that H9N2 virus replication in the oviduct results in poor eggshell quality and deterioration of eggshell [13,15,30]. H9N2 IAV infections in poultry are also associated with co-infections or secondary respiratory pathogens, such as infectious bronchitis virus and *Mycoplasma gallisepticum*, which can lead to high mortality [13,31,32,33,34,35,36,37].

Phylogenetic analyses of the H9 HA have classified the H9 into two lineages: the American and Eurasian lineages, which are further divided into four sublineages (h9.1 to h9.4) [3]. Strains in the h9.1 sublineage are present mostly in wild birds in America, those from the h9.2 sublineage circulate in Korean poultry and wild birds in Eurasia, whereas those from the h9.3 sublineage (BJ94-like strains) are present in poultry in China. Viruses from the h9.4 sublineage (G1-like) are endemic in poultry in the Middle East, India, Egypt and Africa (extensively reviewed in [3]). Antigenic characterization of the HA of H9 subtype IAVs showed that the HA, in particular the globular head, is the immunodominant component, similar to what is described for other IAVs subtypes. Interestingly, the HA of the H9 subtype lacks the 130 lateral loop that forms the antigenic site A, which is an important antigenic region in other subtypes, such as H5 or H3 HAs [38,39]. Such a feature results in two antigenic sites that overlap, designated site I and site II [40]. More recent work showed the presence of alternative non-overlapping antigenic sites designated H9-A and H9-B, where H9-A shares amino acids with site I and is immunodominant in comparison to H9-B [41]. Genetic and antigenic differences are observed within lineages circulating in specific regions, and antigenic drift has been observed in regions where these viruses are endemic, such as China and Egypt [42,43].

Of great significance, H9N2 IAVs have contributed the internal gene segments to more virulent zoonotic strains such as H5N1/N6, H7N9, and H10N8/N3 that have been implicated in human infections and loss of life [44,45,46,47]. In addition, H9N2 IAVs are zoonotic viruses themselves and have also been reported in other mammalian species such as swine, dogs, horses, and mink. In humans, H9N2 IAV infections have presented with mild influenza-like symptoms such as respiratory symptoms, coughing, fever, nasal discharge, sore throat, and headache [48,49,50,51,52]. Only one fatality has been associated with H9N2 IAV infection in humans to date [53]. However, such mild infections could be the prelude to the selection of more virulent strains with the capacity to transmit in humans more efficiently because these mild infections can go unnoticed, allowing the virus to acquire mutations that could increase transmission and replication in humans. Therefore, understanding the factors required for efficient transmission of H9N2 viruses in mammalian species is essential for adequate pandemic preparedness. In this review, we will discuss the transmission of H9N2 IAV in mammalian species, providing an overview of the molecular features that may facilitate the respiratory transmission of these viruses, focusing mostly on the collective findings from our group.

### 1.2. Molecular Mechanisms Associated with Interspecies Transmission of IAVs

**Figure 1:**
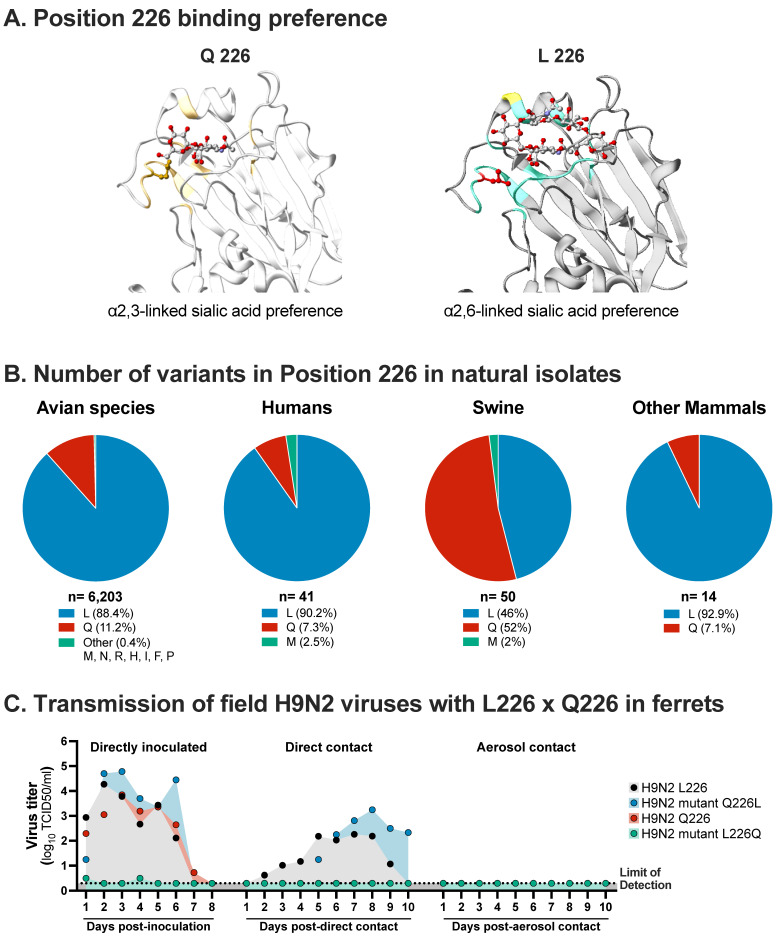
Impact of position 226 in the H9 of H9N2 IAVs. (**A**) The 3D molecular structure of the H9 HA glycoprotein globular head from A/Guinea Fowl/Hong Kong/WF10/99 (WF10) with glutamine (Q) or leucine (L) in position 226. The sialic acid binding pockets are shown in each case (amino acid residues shown in yellow and cyan) with bound sialic acid, shown in gray and red. Structure constructed using the iTASSER structure prediction tool [80]. (**B**) The different residues in position 226 of the H9 were analyzed from H9N2 IAVs isolated for avian species, humans, swine, and other mammals (canine, equine, and mink). Full H9 sequences were downloaded from the Global Initiative on Sharing All Influenza Data (GISAID). Sequence analyses were performed using Geneious Prime 2020.2.4 (https://www.geneious.com, accessed on 19 May 2021). (**C**) Summary of the replication, transmission by direct contact or airborne transmission in ferrets inoculated with H9N2 IAVs viruses carrying L226 (H9N2 L226) or Q226 (H9N2 Q226) in the H9. Data for replication and transmission of mutant viruses with H9 Q226L (H9 mutant Q226L) or H9 L226Q (H9N2 mutant L226Q) are also shown. Results compiled from [71].

Several molecular signatures have been associated with host range restriction and species jump of IAV in mammalian species. Particularly, the segments encoding the HA and the polymerase complex (PB2, PB1 and PA proteins) play a major role in the host range and adaptation of IAVs [54,55,56,57,58,59]. The HA is responsible for receptor-binding to the host cells and the fusion between the endosomal membranes and viral envelope [60,61]. An important barrier in the avian to human transmission of IAV is the different binding specificities of IAV to terminal sialic acid present on the glycan receptors on the host cell surface. IAVs of avian origin bind preferentially to α2,3-linked sialic acids (α2,3SA) which is the most abundant receptor in avian respiratory and intestinal tracts, and those from human origin viruses bind preferentially to α2,6-linked sialic acids (α2,6SA), which is the most abundant receptor in the human respiratory tract [62]. Pigs express both α2,3SA and α2,6SA receptors in their respiratory tract, in a similar distribution to that found in humans, and have been pointed out as a potential intermediary host of influenza viruses [63,64]. The α2,3SA versus α2,6SA preference is mediated by key amino acid residues located in the HA; specifically, positions 226 and 228 (H3-numbering is used throughout the text) are critical for receptor specificity in H3 and H9 viruses (Figure 1A) [65,66,67]. H9N2 IAVs endemic in poultry have variations at position 226, with most isolates carrying leucine (L) and others glutamine (Q) (Figure 1B) [68]. At position 228, glycine (G) is present in almost all H9 isolates detected to date. Indeed, an analysis of more than 2500 H9 isolates from avian and mammalian hosts showed a switch in the 226 position over the years, with the majority of H9N2 IAV isolates carrying Q226 before the year 2000 while newer H9N2 IAV isolates revealed high prevalence of L226 [69]. Viruses with the Q226/G228 combination present dual binding or α2,3SA avian-like preference. A single Q226L mutation produces a switch to α2,6SA human-like preference [70,71]. Consistent with these observations, most H9N2 IAVs identified in poultry farms and live bird markets, which contain L226, exhibit binding to human-like receptors [72,73]. More importantly, most H9N2 viruses isolated from mammalian species, including human isolates, show the L226/G228 combination (Figure 1B). Interestingly, when swine sequences are analyzed, there is an even distribution between isolates carrying Q226 or L226 (Figure 1B).

In addition, other amino acid signatures can modulate and/or enhance binding of H9 HAs to terminal α2,6SAs, such as the mutation from isoleucine (I) to threonine (T) at position 155 [74] or the presence of a valine (V) at position 190 [75]. The modulation of receptor binding preference by V190 is reminiscent of similar effects in HAs of the H1 subtype [57]. The presence of Q227 in combination with either aspartic acid (D) or glutamic acid (E) at position 190 favors binding α2,6SAs receptors. Other changes such as A160D/N, Q156R, T205A, V245I, V216L, D208E, T212I, R1721 and S175N also enhance the human-like receptor binding in vitro [76,77]. The terminal sialic acid linkage is not the only factor that affects the binding of IAV HA to the host cell; other features such as the sialic acid structure (e.g., N-acetylneuraminic acid versus N-glycolylneuraminic acid) or length may also play a role (reviewed in [55]). In addition to specific molecular markers, the pH of fusion may also be an important feature for the mammalian transmission of H9N2 IAVs, since most H9N2 isolates show a low pH of fusion similar to early pdmH1N1 [78,79].

The viral polymerase subunits PB1, PB2, and PA can also contribute to the adaptation of IAV of avian origin to mammalian hosts. Of interest, the change from E to lysine (K) in position 627 (E627K) in PB2 is a major determinant of host adaptation. This has been the predominant host adaptation marker identified in human cases of H5N1 and H7N9 infections [81,82]. A higher frequency of PB2 K627 is observed in H9N2 viruses isolated from mammalian hosts in comparison to their avian counterparts. Interestingly, more than 20% of H9N2 viruses from human cases possess the PB2 V627 signature, which was also observed in transmission experiments between avian and mammalian species [83]. It is worth noting that position 627 modulates the optimal temperature for virus replication. The PB2 K627 is associated with increased polymerase activity and replication at 33–37 °C, an attribute necessary for replication in the human respiratory tract. In contrast, the PB2 E627 mutation allows for optimal replication at 39–41 °C, consistent with the body temperature of most bird species. Furthermore, the PB2 E627K mutation has also been observed 3 days post-inoculation in mice when a duck-origin H9N2 IAV virus is previously serially passaged in chickens and quails, suggesting that the adaptation to land-based birds can also contribute to a faster acquisition of mutations that favor replication in mammals [84], which suggests that PB2 E627K is a respiratory tract adaptation rather than a mammalian adaptation.

The PB2 A588V mutation is also potentially involved in mammalian adaptation. H9N2 IAVs carrying the PB2 V588 signature show enhanced virulence in mice [85]. For PB1, the I368V mutation detected in a ferret adapted H5N1 strain has shown increased frequency among recent H9N2 isolates, from 2.8% to 67% [86]. In PA, the K356R mutation increased viral replication in mice even without PB2 K627. More than 80% of avian H9N2 isolates collected after 2013 and half of human H9N2 isolates contain the PA R356 marker [87]. NA cleavage activity and the interaction of viral ribonucleoproteins (vRNPs) with host restriction factors may also play a role in the interspecies transmission of IAV (extensively reviewed in [55]). A summary of molecular markers associated with transmission and adaptation of H9N2 IAV viruses is presented in Table 1.

### 1.3. Natural Infection of H9N2 IAV in Mammals

According to the World Health Organization (WHO), 58 human cases of H9N2 IAV infection have been reported since December 2015, with 17 of those cases reported during 2021 from the western Pacific region (as of 17 September 2021) [101]. Additional cases have been reported in Bangladesh, India, Egypt, Oman, and Senegal [2,48,102,103,104,105]. Most human infections with H9N2 viruses had confirmed contact with poultry and evidence of human-to-human transmission has not been reported. Serological investigations have shown anti-H9N2 IAV antibodies in humans in Vietnam, Cambodia, Iran, Thailand, Pakistan, India, Egypt, and Hong Kong [106,107,108,109,110,111,112]. In most cases, the presence of anti-H9N2 IAV antibodies is associated with poultry workers; however, there are seropositive cases with no history of direct poultry exposure. H9N2 IAVs have also been isolated from pigs and serological surveys have shown presence of H9N2 IAV-specific antibodies in pig herds with prevalence as high as 15% [52,113,114,115]. In addition, a serological survey showed that minks are also susceptible to infection with H9N2 IAV (31% of positive samples) and six different H9N2 isolates were isolated from tissues obtained from a mink farm. Similar surveys were performed in foxes and raccoon dogs (all species relevant in the fur industry), with 59% and 41% of serologically positive samples, respectively, and H9N2 IAV was successfully isolated in one study [116,117].

### 1.4. Experimental Infections/Transmission of H9N2 IAV in Mammalian Models

For an avian H9N2 virus to successfully transmit in mammals, the virus must evolve to become compatible with the new host environment, allowing effective replication and transmission [62,118]. Reassortment between avian and mammalian influenza viruses has led to the emergence of pandemic viruses in the past. Transmission studies designed to evaluate the potential of IAV transmission in mammals are commonly performed in ferrets, which present similar characteristics for IAV infection as humans in terms of lung pathology, clinical signs, pathogenesis, and immunity [119,120]. Wan and collaborators evaluated the replication and transmission capabilities of different H9N2 IAV isolated from avian species between 1988 and 2003, using the ferret model [71]. Ferrets were intranasally inoculated and the following day, a naïve ferret was placed in the same cage to allow direct contact transmission. At the same time, another ferret was placed in the adjacent cage but within the same isolator allowing for aerosol transmission without direct contact transmission (similar parameters were used in all transmission experiments described in this review). Using this system, it was shown that all the isolates replicated in ferrets. However, just two of those isolates transmitted through direct contact (Figure 1C). The two isolates that transmitted in ferrets contained the L226 HA marker, supporting the advantage of L226 over Q226 for replication in mammals. Regarding the isolates that did not transmit, two had Q226 and one L226. The role of the L226 mutation on mammalian replication and contact transmission was confirmed when it was replaced by Q226 in a H9N2 IAV isolate (L226Q), resulting in complete loss in replication capability, even in the direct inoculated group (Figure 1C). The inverse experiment, Q226L mutation introduced in a virus naturally carrying Q that replicated poorly in ferret without transmission, resulted in enhanced replication and transmission through direct contact (Figure 1C) [71]. Further, transmission studies in ferrets using H9N2 viruses have shown some natural avian-origin H9N2 IAV isolates are able to transmit via respiratory droplets in ferrets without further adaptation, all of which contained the L226 amino acid residue [74,121], highlighting the inherent risk for H9N2 IAV transmission in mammalian species and suggesting a strain-specific effect. Another amino acid residue in the HA, T155, was pointed out as a necessary factor for H9N2 viruses to bind to human-type receptors [74]. Similarly, H9N2 viruses isolated from humans, as well as swine- and avian-origin H9N2 isolates, were able to transmit via direct contact in ferrets, but these included viruses containing both Q226 and L226 [122]. A more recent study showed that different H9N2 isolates from poultry and humans containing mostly L226 exhibited binding to human receptors and replicated efficiently in human bronchial epithelial cells, albeit with delayed kinetics at a lower temperature (33 °C). All human-origin viruses transmitted via direct contact; however, only the two more recent human isolates from the Y280 lineage (containing L226) showed the ability to transmit via aerosol transmission in ferrets [123]. To further understand the relevance of amino acid 226 on HA, the replication of H9N2 viruses containing either L226 or Q226 was tested in human airway epithelial cells grown in an air–liquid interface [66]. Viruses with L226 grew with higher efficiency in comparison with viruses carrying Q226, and showed a different tropism by infecting non-ciliated cells similar to seasonal human H3N2 IAV [66]. In a separated study using ex vivo human respiratory organ culture, H9N2 IAVs were shown to infect both the upper and lower human respiratory tract, with differences observed depending on the strain [124].

More recent work demonstrated that although most H9N2 IAV isolates possess either Q226 or L226, this position is highly flexible and able to tolerate multiple amino acids (at least 10), some not previously detected in natural H9N2 isolates [67]. In vitro, most of these variants replicate to similar titers in comparison to viruses carrying either the Q226 or L226, despite their relative lower receptor binding avidity. Additionally, no impact on antigenicity or hemagglutination activity was observed, independent of the amino acid at position 226. Interestingly, viruses containing N226, M226, or I226 show an increased breadth of receptor recognition, with dual binding to avian- and human-type receptors, a feature that might affect host range and potentially facilitate interspecies transmission. In contrast, viruses carrying Q226, C226, T226 or H226 show strict α2,3 binding, demonstrating a residue-dependency in position 226 for receptor recognition [67]. In vivo competition studies in quails using varying mixtures of these variants demonstrated that the L226 provides a fitness advantage in vivo. A mixture of viruses without Q226 or L226 (varΔLQ) was still able to replicate and transmit via direct contact in quails, granted with lower efficiency at 2 days post-contact (dpc) in comparison with mixtures containing L (var + L), Q (var + Q) or both (var + LQ). Interestingly, sequencing analysis from tracheal swabs showed that even in the varΔLQ or var + Q groups, where viruses with L226 were not included in the mixture, L is still detected in the tracheal swabs collected at 3 days post-inoculation (dpi). Viruses carrying M226 or I226 were also readily detected, consistent with the detection of natural isolates containing such amino acid signatures (Figure 1B). The predominant amino acid detected in contact quails was L226, even in groups in which quail were inoculated with the mixtures lacking L226 (varΔLQ and var + Q), suggesting a strong advantage in transmission when L226 is present. These findings confirm that L226, Q226, and M226 confer a fitness advantage to H9N2 viruses in poultry, explaining their predominance in natural isolates. Although the 226 position has great plasticity, most amino acids result in strains with a preference for α2,3-linked sialic acid and therefore, they are less likely to infect humans. This finding facilitates risk assessment for the zoonotic potential of H9N2 viruses [67].

**Figure 2:**
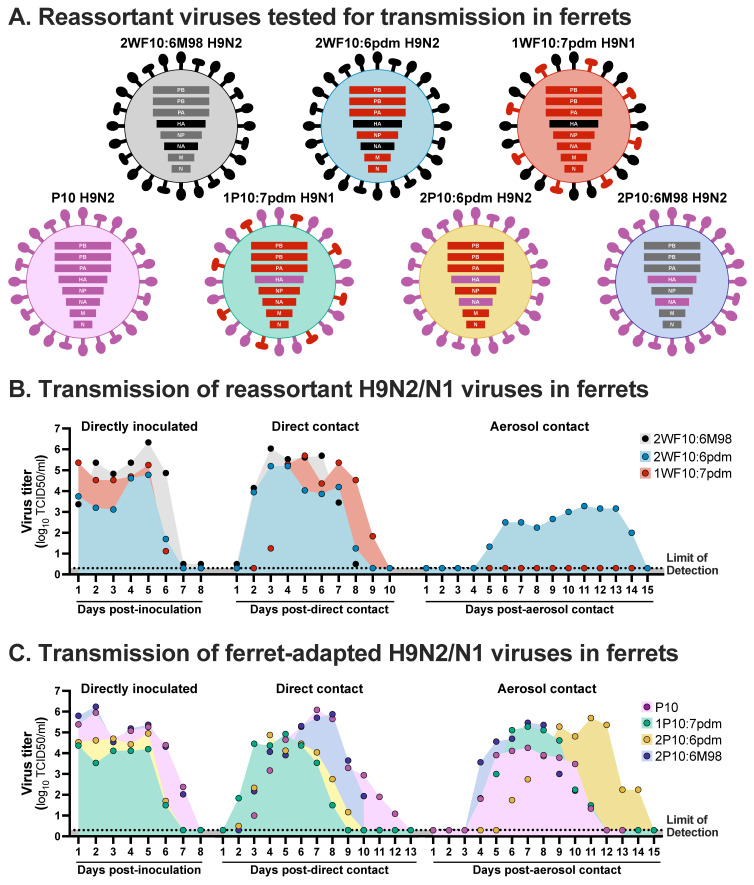
Transmission of reassortant and adapted H9 viruses in ferrets. (**A**) Schematic representation of the different reassortant viruses evaluated in ferrets. (**B**) Replication and transmission by direct contact or airborne in ferrets of viruses carrying the H9N2 subtype with internal genes of a seasonal H3N2 virus (2WF10:M98 H9N2) or pandemic H1N1 virus (2WF10:6pdm H9N2 or 1WF10:7pdm H9N1). (**C**) Replication and transmission by direct contact or airborne in ferrets of a virus carrying the H9N2 subtype with internal genes of a seasonal H3N2 virus adapted by serial passages in ferrets (P10). Viruses containing the HA (1P10:7pdm) or the HA/NA (2P10:6pdm) of the P10 virus and internal genes of a pandemic H1N1 virus, or HA/NA of the P10 virus and internal genes of a seasonal H3N2 (2P10:6M98) were also evaluated. Results compiled from [71,88,98].

Despite direct-contact transmission in ferrets of the H9N2 IAV field isolates in the study described above, no airborne transmission was observed (in contrast to control ferret studies using human origin H1N1 or H3N2 IAVs) [125,126]. Further, ferrets infected with a reassortant virus of the H9N2 subtype with the internal genes of a seasonal human H3N2 strain (2WF10:6M98; Figure 2A) showed clinical signs similar to those observed with the wild type seasonal H3N2 strain. However, direct contact (but not airborne) transmission of the 2WF10:6M98 was observed in ferrets (Figure 2B) [71]. Interestingly, 10 serial passages of the 2WF10:6M98 in ferrets resulted in a virus (P10; Figure 2A) that transmitted efficiently through direct contact and by respiratory droplets (Figure 2C). The P10 virus also showed an intermediate plaque size between the seasonal H3N2 and the H9N2 viruses, suggesting an intermediary replication fitness phenotype [98]. Sequencing of the P10 virus revealed a T189A mutation in the HA1 portion, a G192R mutation in HA2 and a I28V mutation in NA (Table 1). These mutations were crucial for the respiratory transmission phenotype observed. A L374I mutation in PB2 was also detected; however, its contribution seems to be marginal for transmission [98]. After the emergence of the pH1N1 virus in 2009, similar experiments were performed with a reassortant H9N2 virus with pH1N1 internal genes showing that the H9N2-pH1N1 (2WF10:pH1N1) or a H9N1-pH1N1 (1WF10:pH1N1; N1 and internal genes derived from pH1N1; Figure 2A) are able to transmit via direct contact without prior adaptation [88]. These results suggest that in the case of H9N2 IAV, just the introduction of internal genes from human-origin IAV H1N1 or H3N2 is enough to allow an efficient direct-contact transmission in mammals [71,88]. In addition, the 2WF10:6pH1N1 virus was also able to transmit by airborne route, although with delayed replication kinetics, whereas no evidence of airborne transmission with 1WF10:7pH1N1 was observed, suggesting that compatibility and balance between the HA and NA is also important (Figure 2B). It is tempting to speculate that the pH1N1 backbone is more flexible to accept reassortment with diverse surface genes and does provide a replicative advantage in mammals, as has been observed in multiple examples of reassortant H1N1 and H3N2 viruses in swine [127,128,129,130]. Sequencing information showed a S261N mutation in PB1 and V104A in HA in the viruses isolated from respiratory contact animals (Table 1); however, the role of those positions in airborne transmission remains unknown [88]. When pH1N1 internal genes are combined with the H9 HA (1P10:7pH1N1) or H9N2 HA/NA (2P10:6pH1N1) of the P10 ferret-adapted virus (Figure 2A), both viruses show efficient replication in vitro, generate moderate to severe lesions in the respiratory tract of ferrets and both are able to efficiently transmit via direct contact and respiratory droplets in the ferret model [88] (Figure 2C). Furthermore, these viruses efficiently transmitted between pigs by direct contact [99]. Confirming these findings, experimental infection of pigs with field isolates of H9N2 IAV resulted in nasal shedding and seroconversion after infections with four out of six different H9N2 IAV isolates tested [113]; however, transmission between pigs did not occur. In one study, direct-contact transmission of a human-origin H9N2 isolate was confirmed in pigs, although with low efficiency [122]. When the internal gene segments were replaced with those from a pandemic H1N1 strain, increased virus replication and transmission between pigs was observed [131]. Furthermore, when an H9N2 IAV with the pH1N1 internal gene segments was serially passaged in pigs, the virus showed increased tropism capable of replicating in the entire respiratory tract as well as efficient pig-to-pig direct contact transmission [97].

**Figure 3:**
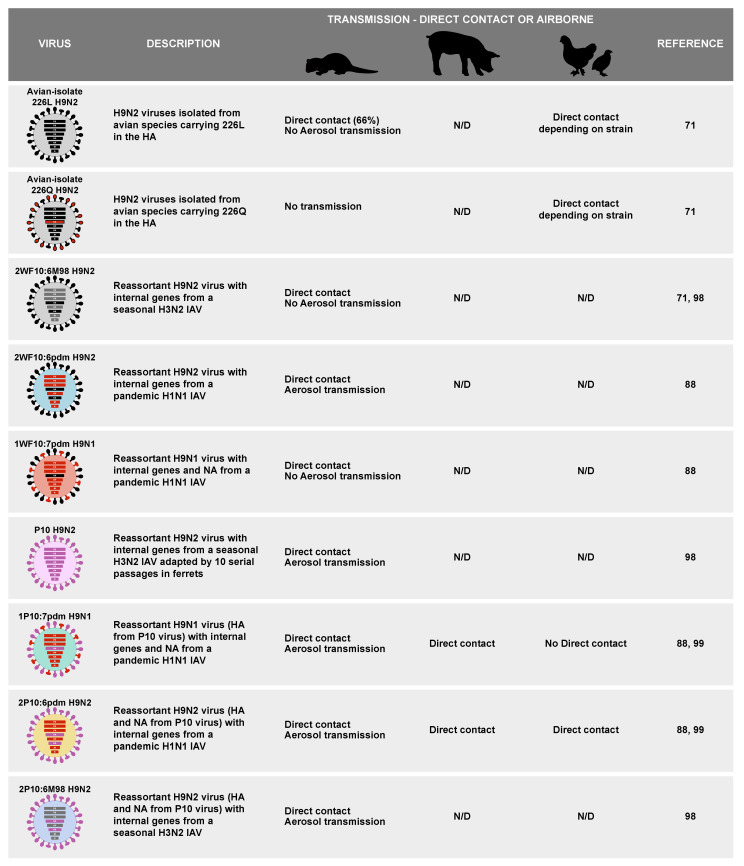
Summary of H9 subtype IAV reassortant viruses and their transmission ability in ferrets. Schematic representation of the different H9 subtype IAVs tested for transmission in ferrets by our group and described in this review, description of each virus, and type of transmission observed. Results compiled from [71,88,98,99].

To confirm the role of reassortment (and presence of mammalian-adapted gene segments) on the ability of H9 viruses to transmit in mammals, a transfection-based inoculation (TBI) study was performed in ferrets to select airborne transmissible H9 reassortant viruses under host selection pressure. In brief, ferrets were inoculated with cells previously transfected with 15 plasmids: 6 encoding the internal gene segments of the WF10 H9N2 virus, 7 gene segments (excluding the HA) of a prototypic pH1N1 virus and the surface gene segments of the P10 ferret-adapted virus. The resulting virus mixture was then serially passaged in ferrets, allowing for the selection of any possible H9 reassortant that was compatible/fit with respiratory droplet transmission in ferrets. The results show two different H9N1 viruses that were selected and able to transmit by the respiratory route in ferrets [90]. Both H9N1 viruses identified had a mixed population of internal genes from pH1N1 or H9N2, both containing PB2, NP and NA from the pH1N1 strain and PA, HA and NS from the H9N2 strain. Both viruses differed in the PB1 and M gene segments where in one virus both were from pH1N1 strain, and the opposite was observed in the second virus. Sequencing analysis revealed mutations in PB2 (D253N), PA (K26E), HA1 (S263N) and NS1/NS2 (D2N) in one of the viruses (Table 1), some of which have been previously reported to have an impact in replication and pathogenicity [89,91,96,100,132]. The second virus showed mutations in PB2 (A707T), HA1 (S328C), M2 (E95K), and two mutations in PB1 (D120N and D439E) (Table 1) [90]. However, the mutations detected in the second virus have not been associated with any advantage in terms of replication or pathogenicity previously, highlighting that the molecular requirements for efficient transmission of H9N2 IAVs in mammalian species are far from understood and deserve further scrutiny. A summary of the different viruses discussed above, and the transmission phenotypes is shown in Figure 3.

Experimental infection/transmission with poultry adapted H9N2 IAV have been evaluated also in multiple alternative animal models such as guinea pigs, mice, and others [71,88,92,98,113,116]. Some H9N2 IAV strains isolated from chicken houses were able to transmit experimentally between guinea pigs by direct contact but respiratory droplets’ transmission was not observed [133]. Interestingly, direct contact transmission efficacy increased after nine serial passages of H9N2 IAV in guinea pigs, reaching 100% transmission after fifteen serial passages, consistent with the idea that molecular adaptative changes are required to result in a mammalian transmissible H9N2 IAV. However, despite the improvement of direct contact transmission after fifteen passages, airborne transmission had only 16% efficacy in guinea pigs and no airborne transmission was observed in ferrets [134]. Similarly, improvement in transmissibility is also achieved in guinea pigs when H9N2 IAV are serially passaged in mice prior to guinea pig infection. The mouse adapted H9N2 IAV has enhanced pathogenicity and is able to transmit through direct contact and respiratory droplets whereas the non-mouse adapted version does not transmit in guinea pigs [92]. Reassortant viruses carrying unmodified H9N2 IAV glycoproteins and internal genes derived from pH1N1 were also able to transmit by either direct contact or via air in guinea pigs whereas a whole avian H9N2 virus did not [135], similarly to what was observed in pigs. The presence of pH1N1 PA gene alone seems to be sufficient to allow transmission between guinea pigs by direct contact but not respiratory droplets [136]. Further, H9N2 IAV isolated from live bird markets possessing L226/S228 in the HA plus K627 in the PB2 (all molecular markers of mammalian adaptation and/or respiratory tissue adaptation) can transmit via air between chickens and guinea pigs [94]. Direct-contact transmission of H9N2 IAV can also occur between minks, foxes, or raccoon dogs [116]. Contact minks developed clinical signs consistent with H9N2 IAV infection whereas seroconversion, but no clinical signs were observed in foxes and raccoon dogs [116]. In another study, the airborne transmission of H9N2 IAV was evaluated in minks, but no positive results were obtained [51]. H9N2 IAV infection was also reported in experimentally infected cats and dogs but transmission through direct contact was only observed among cats but not dogs [137].

## 2. Conclusions

Although H9N2 IAV are LPAIV and cause mostly mild infections, these viruses still result in significant economic losses to the poultry industry. Furthermore, H9N2 IAV have been implicated as the donors of internal genes for prevalent HPAIV outbreaks that have resulted in human infections in some cases. Additionally, cases of H9N2 IAV have been reported in different mammalian species including humans, demonstrating their risk to public health and pandemic potential. Though most infections do not result in mammal-to-mammal transmission, different experiments have demonstrated that transmission of H9N2 IAV in mammals is possible. Nevertheless, adaptation or reassortment with mammalian-adapted internal genes is required for an efficient transmission between mammals, particularly by the respiratory route, although experimental airborne transmission of natural isolates has been observed. Therefore, continued surveillance and research is needed to understand the evolution, pathogenicity, transmission, and antigenicity of H9N2 IAV. Furthermore, understanding the molecular traits that facilitate transmission of H9N2 to and between mammals is crucial to evaluate their pandemic potential and to allow timely identification of viruses with increased potential for interspecies transmission.

## Figures and Tables

**Table 1:** Molecular markers associated with adaptation and transmission of H9N2 IAV in mammalian host.

Protein	Marker	Effect	Host Evaluated	Reference
PB2	T58I	Observed in airborne transmission	Ferrets	[88]
	D253N	Increase pathogenesis/Observed in airborne transmission	Mice and ferrets	[89,90,91]
	R340K	Increase transmission	Guinea pigs	[92]
	K526R	Increase polymerase complex activity and replication	Mice	[93]
	Q591K	Increase polymerase complex activity and replication	Mice	[89]
	E627K	Increase polymerase activity and viral replication in mammalian host	Human, mice, quail, guinea pigs and ferret	[74,81,82,84,93,94]
	A588V	Increase polymerase activity, transmission, and virulence	Mice and guinea pigs	[85,92,93]
	D701N	Increase virulence and airborne transmission	Ferret	[74]
	A707T	Observed in airborne transmission	Ferret	[90]
PB1	D120N	Observed in airborne transmission	Ferret	[90]
	D439E	Observed in airborne transmission	Ferret	[90]
	S261N	Reduced polymerase complex activity/ observed in airborne transmission	Ferret	[88,95]
	I368V	Increase airborne transmission (H5 context)	Ferret	[86]
PA	K356R	Increase polymerase activity and replication	Mice	[87]
	K26E	Increase replication/Observed in airborne transmission	Chickens, quail, and ferrets	[67,90,96]
HA1	D225G	Increase transmission and replication	Pigs	[97]
	Q226L	Increase α2,6SA binding	Ferrets and quails	[67,71]
	I155T	Increase α2,6SA binding	Ferrets	[74]
	A190V/ T190V	Increase replication	Mice	[75]
	V104A	Observed in airborne transmission	Ferrets	[88]
	T189A	Increase airborne transmission	Ferrets, quails, and pigs	[98,99]
	S263N	Observed in airborne transmission	Ferrets	[90]
	S328C	Observed in airborne transmission	Ferrets	[90]
HA2	G192R	Increase airborne transmission	Ferrets, quails, and pigs	[98,99]
NA	I28V	Increase airborne transmission	Ferrets, quails, and pigs	[99]
	A30T	Observed in airborne transmission	Ferrets	[88]
M2	E95K	Observed in airborne transmission	Ferrets	[90]
NS1/NS2	D2N	Increase virulence and IFN-B antagonism/ observed in airborne transmission	Mice/ferrets	[90,100]

## Data Availability

Not applicable.
